# Exometabolomics Approaches in Studying the Application of Lignocellulosic Biomass as Fermentation Feedstock

**DOI:** 10.3390/metabo3010119

**Published:** 2013-02-11

**Authors:** Ying Zha, Peter J. Punt

**Affiliations:** 1 TNO Microbiology & Systems Biology, Utrechtsweg 48, Zeist, 3704 HE, The Netherlands; E-Mail: peter.punt@tno.nl; 2 Netherlands Metabolomics Centre (NMC), Einsteinweg 55, Leiden, 2333 CC, The Netherlands

**Keywords:** exometabolomics approaches, lignocellulosic biomass hydrolysates, inhibitor identification, experimental design, fermentation phenotypes

## Abstract

Lignocellulosic biomass is the future feedstock for the production of biofuel and bio-based chemicals. The pretreatment-hydrolysis product of biomass, so-called hydrolysate, contains not only fermentable sugars, but also compounds that inhibit its fermentability by microbes. To reduce the toxicity of hydrolysates as fermentation media, knowledge of the identity of inhibitors and their dynamics in hydrolysates need to be obtained. In the past decade, various studies have applied targeted metabolomics approaches to examine the composition of biomass hydrolysates. In these studies, analytical methods like HPLC, RP-HPLC, CE, GC-MS and LC-MS/MS were used to detect and quantify small carboxylic acids, furans and phenols. Through applying targeted metabolomics approaches, inhibitors were identified in hydrolysates and their dynamics in fermentation processes were monitored. However, to reveal the overall composition of different hydrolysates and to investigate its influence on hydrolysate fermentation performance, a non-targeted metabolomics study needs to be conducted. In this review, a non-targeted and generic metabolomics approach is introduced to explore inhibitor identification in biomass hydrolysates, and other similar metabolomics questions.

## 1. Introduction

In the last decade, more and more attention has been paid to using lignocellulosic biomass as feedstock for bulk chemical production with biotechnology processes [1,2]. This biomass, including for example wheat straw, corn stover and bagasse, consists mainly of agricultural residues, which is renewable and not competitive with world food supply [3,4]. If microorganisms could use such biomass efficiently as fermentation feedstock, production processes would be less expensive and more environmentally friendly. 

**Figure 1:**
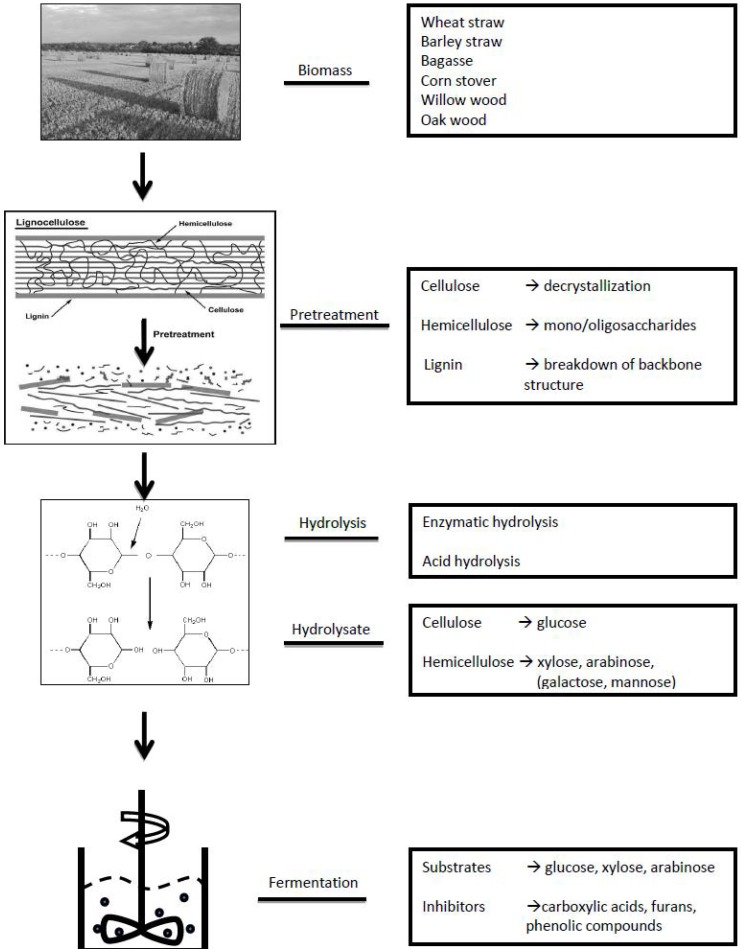
Schematic workflow for the preparation of lignocellulosic biomass hydrolysates and their use in microbial fermentation. Details of the approach are described in the text (paragraphs 1 and 2).

Lignocellulosic biomass is mainly composed of cellulose, hemicellulose and lignin (Figure 1). Cellulose is a polysaccharide consisting of D-glucose, and it forms the backbone structure of lignocellulose; hemicellulose is composed of a matrix of different polysaccharides, such as xylan, arabinoxylan and xyloglucan; in addition, lignin is a complex aromatic polymer, functioning as the supportive structure of lignocellulose [5,6]. Due to the rigid structure of lignocellulosic biomass, very few microorganisms can use the biomass directly for growth and production. Therefore, prior to feeding the biomass into fermentors, a pretreatment-hydrolysis step is carried out to break down the structure of lignocellulosic biomass and hydrolyze the exposed polysaccharides into monomers [7,8]. The conditions under which feedstock is pretreated are quite harsh, involving high temperature, high pressure and an acidic/alkaline environment [9,10,11]. Pretreatment not only results in the disruption of the lignocellulose structure but also in the formation and release of compounds, which could negatively influence the fermentation processes. Therefore, when biomass hydrolysates (hydrolysis products of lignocellulosic biomass) are used as fermentation media, their fermentability by microbes is reduced compared to synthetic media with pure sugar monomers as carbon source (Figure 1) [12,13]. 

To identify and ultimately reduce the effects of inhibitory compounds on the fermentation processes, insight into biomass hydrolysate composition and its relationship with fermentation performance is required. One way to obtain this insight is through a so-called metabolomics approach. That is, by studying the relationship between (the change of) metabolite levels and performance of the biological system [14,15]. 

Metabolomics is a functional genomics approach aimed at studying the diversity of biological systems by analyzing intra- and extra-cellular metabolites. Compared to genomics, transcriptomics and proteomics, metabolomics reflects most directly the physiological status of a biological system, as metabolites links most closely to the phenotype of an organism [15,16]. In the last two decades, a diverse range of techniques that can detect and quantify metabolites with various properties have been developed. Metabolomics has been applied in the areas of pharmacy, food and nutrition, plant research and biotechnology [17,18]. Metabolomics studies include detecting metabolite level change caused by genetic modification and/or altered environmental conditions [19,20], finding bio-makers that improve the performance of a biological system [21], and sample classification [22]. 

Metabolites are small organic compounds participating as intermediates or products in metabolic pathways. Metabolites that are secreted into fermentation media are defined as exo-metabolites together constituting the so-called exo-metabolome. As the chemical properties of different metabolites are diverse, usually several different analytical techniques are required to conduct a metabolomics study [16,23,24]. 

Different metabolomics approaches may be adopted, such as metabolite target analysis, metabolite profiling, metabolomics and metabolic fingerprinting [14]. With metabolite target analysis and metabolite profiling, a selection of metabolites is made based on previous research and expert knowledge, and for the most part a single analytical technique is chosen for measuring this group of compounds. These approaches allow a simple sample analysis process and avoid dealing with complex data-sets. However, though widely applied, these approaches are often biased, neglecting the metabolites that are not in the selection. This can artificially amplify effects of selected compounds on the performance of the biological system, losing information like synergetic effect with compounds not selected for analysis [25]. When it is not known which metabolites are of importance in the research question, a non-targeted metabolomics approach becomes essential, since the approach does not involve compound pre-selection. 

Metabolomics approaches, mostly targeted, have been used to study the composition of lignocellulosic biomass hydrolysates, in relation to their performance as fermentation media. The “exo-metabolites” in such metabolic footprinting studies are components of biomass hydrolysates [26]. These exometabolomics studies help to identify compounds that inhibit the growth of fermenting microbes, reveal the dynamics of some inhibitory compounds in detoxification and fermentation processes, and provide evidence to optimize pretreatment conditions. To further investigate the overall composition of different types of biomass hydrolysates, and study potential inhibitors in these hydrolysates unbiased, a non-targeted exometabolomics approach should also be adopted. 

In this review, we present several targeted exometabolomics approaches with which the composition of lignocellulosic biomass hydrolysates was studied. The analytical methods used for analyzing the non-sugar compounds in biomass hydrolysates are summarized. The use of targeted approaches in improving pretreatment conditions and fermentation performance of hydrolysates is illustrated. Furthermore, a non-targeted and generic exometabolomics approach is introduced. The approach is applied to identify inhibitors in different types of biomass hydrolysates unbiased and to study their dynamics in fermentation processes.

## 2. General Approach of Metabolomics Studies

**Figure 2:**
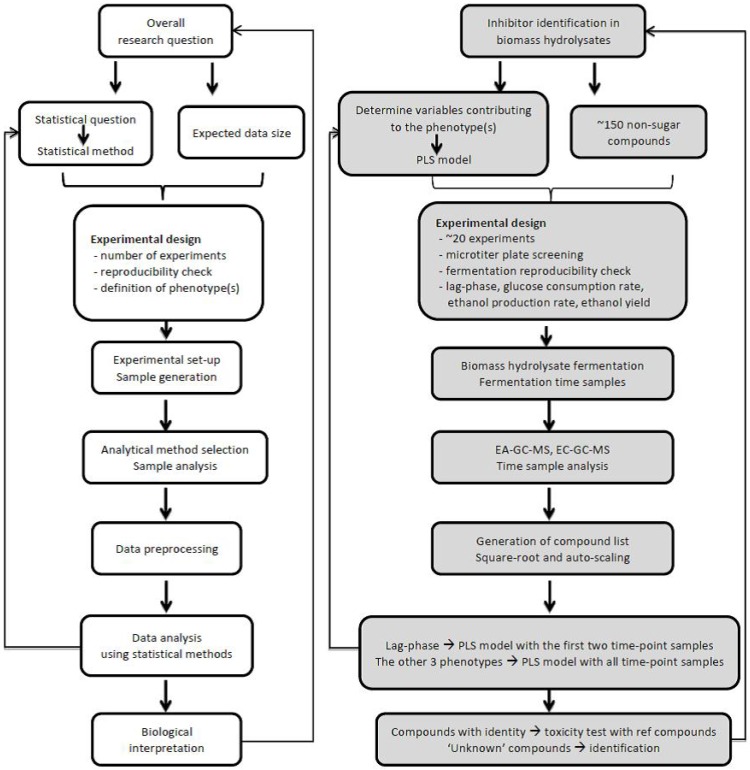
(**A**) Overview of a general metabolomics workflow, (**B**) Non-targeted metabolomics workflow used for studying the use of lignocellulosic biomass hydrolysate as fermentation medium in particular by identifying inhibitory compounds. Details of the approach are described in the text (paragraphs 2 and 4).

In general, the goal of a metabolomics study is to address biological questions by measuring relevant metabolites in a biological system. The measured metabolites are used to reveal their relationship with the performance of the biological system through statistical means. A flowchart illustrating the general metabolomics approach is shown in Figure 2A. 

The first step is to define a research question that clearly describes the aim of the study. The question should be informative and specific, pointing out both the analytical targets and the biological system of the study [27]. When the research question is clear and specific, it can be translated into a statistical question, based on which experimental design is carried out and tentative statistical methods are chosen.

Based on the defined research question, an estimation of the amount of metabolites to be measured can be made. The number of metabolites to be measured relates not only to the property of the biological system, but also to the coverage of the analytical methods used. For instance, when both GC-MS and LC-MS were used to analyze the metabolome of *Escherichia coli*, the detection of between 250 and 500 metabolites was estimated [16]. Based on the number of metabolites to be analyzed, the number of different experiments can also be determined. The larger the number of metabolites, the more experiments should be carried out to acquire a reliable answer to the research question [28,29,30]. 

Knowing the required number of different experiments to be conducted, experimental design can be performed. The requirements of the designed experiments are that they (1) closely relate to the research question, (2) reflect real-life situations, and (3) result in a range of well-spread measurable phenotypes [31,32]. To ensure the success of the experimental design, information about the operability and repeatability of the experiments needs to be obtained beforehand. Preferably, more experiments than desired are initially conducted, so that, when certain experiments do not meet the requirements mentioned above, they can be discarded from the design. 

Another key point in experimental design is defining the phenotype(s) to characterize the performance of the different experiments. Depending on the selected phenotype, experimental set-up and sampling strategies will be determined. Phenotypes are parameters defined to describe the research question in a quantitative manner. There is no universal phenotype definition, since the focus of each study is different, and often more than one phenotype is needed to fully represent the research question. The importance of defining proper phenotypes and its influence in answering the research question are illustrated by Braaksma *et al.* [19]. In her study on enzyme production by the filamentous fungus *Aspergillus niger*, six different phenotypes were defined to be able to fully address the research question. In addition, different metabolite target groups were found to be correlating to different phenotypes. Therefore, defining a series of phenotypes that address different aspects of the research question is strongly recommended. 

As soon as phenotypes are defined, experimental process and sampling can be set up to obtain parameters needed to calculate the phenotypes. For practical reasons, it is preferred to set up as simple an experimental process and sampling method as possible, given that all necessary parameters can be acquired. One should bear in mind that the sampling method is also determined by the biological system and the sample analysis techniques of the study. The sample work-up of extracellular metabolomics (exometabolomics) is much simpler than intracellular metabolomics, which needs sample quenching, metabolites extraction and biomass correction [25,26]. When GC-MS is used to analyze samples, extra sample preparation steps, like derivatization, are often required, which is generally not required for LC-MS and NMR analysis [23].

In targeted metabolomics, analytical methods are chosen based on the properties of the pre-selected compounds. In non-targeted metabolomics, analytical techniques also need to be chosen, as it is not possible to use all available methods for sample analysis. Besides, it is more informative to focus on metabolite classes that are relevant to the aim of the study. Therefore, analytical methods in non-targeted metabolomics should still be selected based on the research question and known properties of the biological system. For instance, when it is known that volatile compounds may be important to the research question, methods allowing the analysis of these compounds, such as solid-phase microextraction (SPME), should be used [33,34]; and as the focus of the study is on carbohydrates, methods like high-performance anion-exchange chromatography with pulsed amperometric detection (HPAEC-PAD) or LC-MS should be selected [35,36].

In non-targeted metabolomics, sample analysis results in a list of detected compounds, both known and unknown, and their relative quantities, presented as peak areas in chromatograms. The analysis results of all samples in the experimental design form a data-set, which will be studied statistically. Before the data-set is analyzed statistically, it needs to be preprocessed. Generally, data preprocessing involves the following aspects, (1) peak area correction with internal standards, (2) data-set normalization, and (3) data-set transformation. Peak area correction is conducted to minimize the influence of sample matrix, an effect caused by the overall composition of the sample; normalization is carried out to reduce the redundancy of the data-set; and transformation is performed to increase the useful information content the data-set carries. There are multiple ways to preprocess a data-set, and the methods chosen are specific to the analytical technique used and the statistical model selected. Detailed discussions on data preprocessing are given by Roessner *et al.* [37] and van den Berg *et al.* [38].

To find the relationship between the preprocessed data-set and the defined phenotypes in non-targeted metabolomics study, multivariate data analysis (MVDA) tools are applied. The most commonly used tools are principal component analysis (PCA), partial least square (PLS), and discrimination/classification methods. PCA model points out variables (metabolites) that contribute the most to the data-set structure [39]; PLS model seeks metabolites that are most responsible for a certain phenotype [40]; discrimination/classification methods determine if a sample belongs to a specific group [28]. Based on the research question, one or several of the MVDA tools are selected to analyze the preprocessed data-set. Two other factors to be considered when conducting MVDA are 1) fusing of the data-sets generated by different analytical methods and its influence on the model building results, and 2) methods for model validation. Simply using MVDA tools for analyzing metabolomics data-sets without checking the validity of the models can produce misleading or even wrong results. Rubingh *et al.* addressed the complexity of the real-life metabolomics data. Several model validation methods were provided to attain more reliable and comprehensive data analysis results [29]. 

Compared to non-targeted metabolomics, the compound list in a targeted approach is very short. Since the compounds are pre-selected, their absolute concentrations can be determined with reference compounds. This simplifies or even omits data preprocessing, and makes data analysis straightforward and simple.

The last step in a metabolomics study is to translate the statistical analysis results into the biological context to answer the research question. Some analytical results speak for themselves, like the ones in discrimination/classification studies [41], while others are complex, especially those involving metabolites identification [42]. There are several tools that assist the biological interpretation, which are illustrated by van der Werf *et al.* [25]. Additionally, it should be noted that non-targeted metabolomics analysis might suggest compounds that seem to be ‘incorrect’ based on expert knowledge. They are either not previously found in any similar biological systems, or known to function in an unrelated biological process. Such compounds should also be taken into account for future research, since they may play a role in further understanding the biological system studied.

## 3. Targeted approach: Applying targeted Metabolomics Approaches to Study the Sugar and Lignin Degradation Products in Lignocellulosic Biomass Hydrolysates

Most of the targeted approaches start with analyzing the structure of lignocellulosic biomass, which reveals several main degradation products in biomass hydrolysates, the pretreatment-hydrolysis product of lignocellulose. As shown in Figure 1, cellulose, hemicellulose and lignin are the three main components of lignocellulosic biomass. Cellulose is the linear polymer of β-1,4-linked D-glucose residues, hemicellulose is a heteropolymer mainly containing xylan, arabinoxylan and xyloglucan, when hydrolyzed generating xylose, mannose, galactose, arabinose and glucose [43]. Lignin is a complex macromolecule composed of phenylpropane units, which are the dehydrogenation products of *para*-coumaryl alcohol, coniferyl alcohol, and sinapyl alcohol [13]. The degradation products of the sugar monomers of cellulose and hemicellulose, and lignin are generally categorized into small carboxylic acids, furans and phenolic (aromatic) compounds [12,44]. Formic, acetic and levulinic acid are the most common small carboxylic acids, while furfural and 5-hydroxy-methylfurfural (HMF) are the representatives of furans [45]. Comparatively, the diversity of phenolic (aromatic) compounds in biomass hydrolysates is much greater [46,47,48,49]. In this section, the analytical methods used to detect and quantify these three categories of compounds are presented. Furthermore, application of targeted metabolomics approaches on identifying inhibitors in biomass hydrolysates and improving hydrolysate preparation methods is reviewed.

### 3.1. Analytical Methods for Studying Hydrolysate Composition

As many of the targeted studies referred to in this review are focused on specific classes of compounds, analytical methods used to detect and quantify these are discussed separately. However, general aspects of these analytical tools are often not specific for the compound classes.

#### 3.1.1. Small Carboxylic Acids and Furans

Several methods have been extensively used to detect and quantify small carboxylic acids and furans in biomass hydrolysates, among which are High-Performance Liquid Chromatography (HPLC), and Capillary Electrophoresis (CE) (Table 1). 

**Table 1:** Analytical methods used for detecting compounds in lignocellulosic biomass hydrolysates.

Analytical method	extraction / derivatization	Detected compounds	Identification	quantification	reference
HPLC	no	formic, acetic acid, levulinic acid, lactic acid, glycolic acid, malic acid, citric acid, succinic acid, oxalic acid	no	yes	[49,50,51,55,58,59,60,61,62]
		furfural, HMF furfuryl alcohol, 2,5-bis-hydroxymethylfuran			[49,55,58,60,61,63,64]
RP-HPLC	precipitation-filtration, MTBE / no	formic acid, lactic acid, acetic acid, levulinic acid, furfural, HMF, phenolic compounds	partial	yes	[52]
	MTBE / no	gallic acid, furfural, HMF, protocatechuic acid, vanillin, coniferyl alcohol, syringaldehyde, sinapic acid	partial /GC-MS		[51]
	no	reference phenolic compounds	GC-MS		[50]
CE	no	formic acid, acetic acid, levulinic acid, glycolic acid, lactic acid, furfural, HMF	no	yes	[53,63,65]
GC-MS	solvent / no	acetic acid, furfural, acetamide	no	yes	[66]
	MTBE / silylation	gallic acid, HMF, vanillin, protocatechuic acid, syringaldehyde	yes/partial	no	[51]
	DCM / EC-derivatization	levulinic acid, furfural, furfurylalcohol, 2-furanmethanol acetate, HMF, phenolic compounds		yes	[67]
	SPE / silylation	phenolic compounds			[60,61]
	EA / silylation	furfural, HMF, furfuryl alcohol, 2-furoic acid, phenolic compounds			[55]
		phenolic compounds		no	[49]
		phenolic compounds		yes	[57,59]
	no / silylation	lignin derived monomer and dimers			[65]
LC-MS/MS	precipitation-filtration, MTBE / no	aliphatic acids, furans, phenolic compounds	yes	yes	[54,68]

MTBE: methyl tertiary butyl ether; DCM: dichloromethane; SPE: solid phase extraction; EC: ethylchloroformate; EA: ethylacetate.

HPLC is the most standard method for quantifying monomer sugars, simple small carboxylic acids, furfural and HMF, though the analytical system and column used may vary (Table 1). The method requires little sample work-up and detects a limited range of target compounds, which are quantified by making calibration curves using external standards. RP-HPLC is a variation of HPLC that detects a much larger group of compounds with identification possibility only when followed up by GC-MS [50,51]. RP-HPLC assigns identity to detected compounds mainly by comparing their retention time to and/or spiking samples with reference compounds. In the identification process, no compound structural analysis is involved and the availability of reference compounds is a necessity. Therefore, the identification conducted by RP-HPLC requires prior knowledge [52].

It can also be seen from Table 1 that an extraction step using methyl tertiary butyl ether (MTBE) is often used before analyzing hydrolysate samples with RP-HPLC in combination with detection based on refractive index (RI). This is because hydrolysate samples normally contain high concentrations of sugars, like glucose. These huge sugar peaks appear in RP-HPLC chromatograms interfere with the RI detection of target compounds, like furans. Therefore, to minimize the disturbance, sugars are removed by extracting hydrolysates with organic solvent before conducting analysis. This applies also to GC-MS method, which requires an extraction step before the derivatization step in sample preparation (Table 1). Besides small carboxylic acids and furans, phenolic (aromatic) compounds can also be studied by RP-HPLC. This will be discussed in the next section. 

CE is yet another method for analyzing the described compounds in hydrolysates. Compared to RP-HPLC, the targets of CE are more specific, mainly small organic acids. Like in HPLC, little sample work-up is needed for CE, and the method cannot be used for identification of novel compounds. When analyzing hydrolysate samples, it is preferred to measure both carboxylic acids and furans with one analytical method. Since HPLC is capable of detecting both acids and furans, the method is often chosen above CE. Recently, it was shown that CE can also separate saccharides and furans in hydrolysate samples, and the quantification results of CE on furfural and HMF are highly comparable to HPLC [53]. Therefore, CE has the potential to become a routine analytical method for measuring hydrolysate samples. 

#### 3.1.2. Phenolic (aromatic) Compounds

As addressed before, phenolic (aromatic) compounds are mostly the degradation products of lignin, and due to the complexity of lignin structure, the chemical structure of this group of compounds in biomass hydrolysates is very diverse. The potential phenolic compounds in hydrolysates derived from the three basic lignin building blocks, namely *para*-hydroxyphenyl (H), guaiacyl (G), and syringyl (S) residues, are summarized by Klinke *et al.* [13] (Table 2). It was estimated that about 60 different phenolic compounds could be found in various hydrolysates, including compounds with unknown structures.

**Table 2:** Phenolic (aromatic) compounds detected in the studies listed in Table 1.

*p*-hydroxyphenyl residue (H)	Detected in more than one study*	Detected in one study	hydrolysate	ref
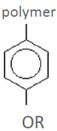	phenol 4-hydroxybenzaldehyde 4-hydroxybenzoic acid salicylic acid (2-hydroxybenzoic acid) 3,4-dihydroxybenzaldehyde benzoic acid catechol (1,2-dihydroxybenzene) *p*-coumaric acid (4-hydroxycinnamic acid) piceol (4-hydroxyacetophenone)	hydroquinone	spruce-dilute acid	[60]
		4-methoxyphenol *p*-coumaryl alcohol **Phloretic acid** (3-(4-hydroxyphenyl)propionic acid)	wheat straw-steam explosion	[55]
		*o*-cresol (2-methylphenol) gentisic acid (2,5-dihydroxybenzoic acid) protocatechuic acid (3,4-dihydroxybenzoic acid)	willow-acid steam	[57]
		caffeic acid (3,4-dihydroxy cinnamic acid)	corn stover-dilute acid or ammonia fiber expansion	[54]
Guaiacyl residue (G)				
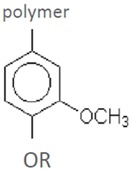	guaiacol vanillin vanillic acid homovanillic acid ferulic acid (4-hydroxy-3-methoxycinnamic acid) 3-hydroxy-4-methoxycinnamic acid coniferyl aldehyde dihydroconiferyl alcohol acetovanillone (acetoguaiacone)G-CH2COCH3	G-CHOHCOCH3 G-COCOCH3 G-CH2COCH2OH G-COCHOHCH3	spruce-dilute acid	[60]
		vanillyl alcohol G-CH2CH2COOH G-CHCHCHO	wheat straw-steam explosion	[55]
		*trans*-isoeugenol	willow-acid steam	[57]
Syringyl residue (S)				
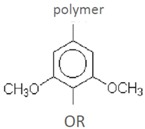	**syringaldehyde** **syringic acid**	acetosyringone	wheat straw-alkaline wet oxidation	[61]
		syringol S-CHCHCHO	wheat straw-steam explosion	[55]
Other structures		biphenyl-type dimer diarylpropane-type dimer pinoresinol-type dimer	Japanese beech-hot compressed water	[65]
		4-hydroxycoumarin *o*-toluic acid *p*-toluic acid	corn stover-dilute acid or ammonia fiber expansion	[54]

* The compounds listed in this column appeared in two or more studies listed in the “ref” column and the following three references: [49] [52] [67]. The hydrolysates used in these three studies were corn stover-dilute acid, yellow poplar organosolv, and bagasse and oak hydrolysates.

To detect, identify and quantify these phenolic compounds in hydrolysates, several different methods have been applied, including RP-HPLC, Gas Chromatography–Mass Spectrometry (GC-MS) and Liquid Chromatography–Mass Spectrometry^2^ (LC-MS/MS), see Table 1. A common characteristic of the three techniques is that they all possess the possibility of (partial) identification, which is essential for studying a diverse group of lignin degradation products with many ‘unknowns’. 

Compound identification with RP-HPLC and LC-MS/MS is mainly done by first constructing chromatograms with a relative large group of reference compounds. The generated chromatograms are then compared with the peaks in the sample chromatogram [52,54]. By comparing the retention time in LC and/or extract mass information provided by MS, identities can be assigned to peaks in hydrolysate samples. Since for each identified compound, its reference is already available, quantification can be directly carried out by generating calibration curves. 

In contrast to LC methods, GC-MS appears to be more open, as reference compounds are only involved in a later stage of the analysis. The initial identification with GC-MS is often conducted by comparing compound fragment profiles with a mass spectral library [48,55,56]. In some cases, reference compounds are used to confirm the identity of characterized peaks [48]. Even when identity is not assigned, an indication of the category the compound belongs to can be given [57]. Therefore, GC-MS seems to be a preferred method for studying phenolic (aromatic) compounds in biomass hydrolysates. The method has been adopted in multiple studies, resulting in the identification and quantification of a variety of phenolic (aromatic) compounds, see Table 2. It can be seen that most identified phenolic compounds fell into the categories of the three lignin building blocks, primarily aldehyde and acid forms. More derivatives of *para*-hydroxyphenyl residues (H) were found than guaiacyl derivatives (G) than syringyl derivatives (S) (Table 2). Phenolics dimers and non-phenolic aromatic compound, namely toluic acid, were also detected. These analysis results confirmed that there is a diverse group of phenolic compounds in biomass hydrolysate, indicated that the phenolic compound composition in different hydrolysates vary. 

### 3.2. Application of Targeted Approaches in Studying Biomass Hydrolysates

The detection and quantification of the degradation products of sugars and lignin in lignocellulosic biomass hydrolysates not only revealed the presence and level of such compounds, but also provided information to (1) test the toxicity of these compounds towards microbes, (2) study the formation conditions of these compounds, (3) trace their dynamics in a detoxification treatment or during a fermentation process. The applications of targeted metabolomics approaches on studying these aspects of biomass hydrolysates are discussed below.

#### 3.2.1. Inhibitor Identification

Biomass hydrolysates generated from different pretreatment methods exhibit inhibitory effects when used as fermentation media. Some elongate lag-phase, some reduce growth rate, some lower product yield, while others abolish growth completely [12,44]. The inhibitory effects are the results of compounds present in hydrolysates, which are formed or released during the pretreatment process. These inhibitory compounds are mostly sugar and lignin degradation products, which can be different in each hydrolysate. To improve the fermentability of biomass hydrolysates, identifying these compounds is crucial. The identification has been carried out by using targeted metabolomics approach.

Most studies start with selecting a group of compounds that are potentially inhibitory in biomass hydrolysates. The selection was made based on expert knowledge as well as previous research results. For instance, in the study of Chen *et al.* [52], aliphatic acids, phenols, aromatic acids and aromatic aldehydes were selected as they were reported as major degradation products in biomass hydrolysates [13]. According to the chemical properties of the selected compounds, analytical methods were established to measure and, in some cases, quantify these compounds. Both RP-HPLC and GC-MS have been used in such studies, and pure reference compounds were used for both identification and quantification purposes [50,52,59]. In some studies, the presence of the selected compounds in the actual hydrolysate was checked [52,58], while in other studies, their inhibitory effects towards one or several microbes were tested by spiking with various concentrations [50,69]. 

In some other studies, the pre-selection of potential inhibitors was not conducted, hydrolysates were typically analyzed with GC-MS, and the mass spectra of the resulting peaks were used for compound characterization [49]. The characterization was either done by comparing the mass spectra of the detected peaks to a mass spectral library [48,55,56], or comparing them to a series of reference compounds [51,59]. When a mass spectral library is used, a large group of compounds can be characterized [55]. However, instead of exploring the inhibitory effect of each detected compound, the authors decided to focus on vanillin and furfural based on previous research results. This makes such a study targeted from this point on. Compared to approaches using reference compounds, the benefit of directly analyzing hydrolysates with GC-MS is that as soon as the compound is characterized, its presence in the hydrolysate is also confirmed. The concentration of the characterized compound can be determined with its reference compound, and its toxicity can be tested according to its concentration present in the hydrolysate [51,67]. 

#### 3.2.2. Pretreatment Condition Optimization

It is known that the inhibitory compounds in biomass hydrolysates are mainly formed during pretreatment process, which is in most cases operated under harsh conditions (Figure 1) [9,10,11]. The fermentability of a specific hydrolysate is, to a great extent, determined by its pretreatment [11]. Thus, studying the relationship between biomass pretreatment and its resulting hydrolysate composition provides valuable information for selecting appropriate pretreatment conditions. 

A targeted metabolomics approach has been used to study the influence of pretreatment conditions on fermentable sugars and inhibitors formation of a specific pretreatment method [62,63]. The approach started with designing experiments by varying specific pretreatment conditions, such as temperature and residence time, both individually and together. All different pretreatment conditions were quantitatively represented by a series of combined severity factors (CS), and under each CS, a pretreatment experiment was carried out. Samples were taken from the resulting hydrolysates of different CS for analysis. The fermentable sugars and inhibitors to be analyzed were pre-selected based on expert knowledge, which in turn determined the analytical methods. As the inhibitors selected in these studies were small carboxylic acids, furfural and HMF, HPLC and CE were used to quantify these compounds in the hydrolysate samples (Table 1). Based on the analysis results, the authors evaluated the influence of CS on the formation of fermentable sugars, as well as on the release of the selected inhibitors, which provided criteria for choosing the optimal pretreatment conditions. 

A similar approach has been applied by Klinke *et al.* to not only determine the optimal pretreatment conditions, but also study the correlation between pretreatment conditions and the degradation products [61]. In such a study, a much larger range of potential inhibitory compounds were selected, which included not only carboxylic acids and furans, but also phenolic compounds. Hydrolysates, prepared at different pretreatment conditions, were analyzed with GC-MS for their phenolic contents. The identification of the phenols was conducted by comparing their MS spectra with a mass spectral library, and standards were used to verify the identity and quantify these compounds in hydrolysates. The correlation between pretreatment conditions and the detected degradation products was studied statistically, using principal component analysis (PCA), revealing the influence of each single pretreatment condition on the formation of degradation products. 

#### 3.2.3. Monitoring Compound Dynamics during Detoxification and Fermentation

To reduce the toxicity of biomass hydrolysates as fermentation media, detoxification methods have been developed to remove inhibitors in hydrolysates [44,70]. The effects of detoxification were improved fermentability and increased product yield [71,72,73]. To study beyond the effect of hydrolysate detoxification, the composition change in terms of (potential) inhibitory compounds in hydrolysates needs to be monitored during the detoxification. Such studies were conducted using targeted metabolomics approaches. The most straightforward way of studying a detoxification process was by using the already identified inhibitors as monitoring targets. These inhibitors mainly include small carboxylic acids, furfural and HMF. Typically, the concentration of these compounds was determined before and after the detoxification process, using HPLC [72,74,75]. The targets of each detoxification method can be different, as far as monitored compounds were considered. For instance, it was discovered that the chemical detoxification by overliming was specifically effective to furans [74,76]. In the study of Martinez *et al.* [76], besides the selected inhibitors, the authors also looked at the unknown peaks in the HPLC chromatogram. Among those unknown peaks, three decreased after overliming, indicating that more compounds could be involved in resulting the detoxification effect of this specific method.

When the detoxification targets are neither small carboxylic acids nor furans, a different targeted metabolomics approach than the one discussed above should be applied. In the case of enzymatic detoxification using laccase, phenols were assumed to be the detoxification targets, as laccase is a phenol oxidase. This assumption was verified by Larsson *et al.* [60] through quantifying small carboxylic acids, furans and total phenols in spruce hydrolysate. To study the detoxification effect of laccase on individual phenolic compounds, both HPLC and GC-MS were adopted [56,57,77]. When HPLC was used, a pre-selection of phenolic compounds was made based on the reported toxicity of these compounds, and their detectability by HPLC [77]. When hydrolysates were analyzed with GC-MS, the compound pre-selection was not done. The phenols detected by GC-MS were characterized either by comparing to a mass spectral library [56] or using reference compounds [57]. The advantage of using GC-MS is that the relative quantity of some unidentified compounds can also be determined to check if they were (partially) removed from the hydrolysate after detoxification.

Similar to detoxification, it was observed that during a fermentation process, the hydrolysate toxicity reduces. This is because the fermenting microbe can transform inhibitors to their less toxic form [45,78]. Targeted metabolomics approach also contributed to study the chemical conversion of these compounds. In such studies, the identified inhibitors were taken out of the context of hydrolysates and added into synthetic medium for growth testing. The conversions of these compounds were predicted based on expert knowledge, and analytical methods were selected accordingly. The conversion of furfural and HMF were monitored by analyzing their alcohol forms during fermentation processes with HPLC [64,79,80]. In addition, the conversions of vanillin and coniferyl aldehyde were investigated with RP-HPLC and GC-MS [50]. To examine these conversions in hydrolysates, GC and GC-MS were used to monitor different forms of furan and phenolic compounds, namely aldehydes, alcohols, ketones, and acids [67,81]. Similar trends of conversion from aldehyde to alcohol and acid form were observed in hydrolysates, though their quantitative relationships were not as straightforward as those in synthetic medium. These results suggested that aldehydes are more likely to be the inhibitory forms of furans and phenols in biomass hydrolysates. 

By monitoring the dynamics of above mentioned compounds during detoxification and the fermentation process, it was shown that all three groups of proposed inhibitors could negatively influence hydrolysate fermentability. Especially for phenolic compounds, their toxicity was confirmed both in the laccase study and by their conversions during fermentation processes. Phenolic compounds have much greater diversity in hydrolysates compared to small carboxylic acids and furans. The overall composition of phenolic compounds was hardly studied in relation to their toxicity in biomass hydrolysates. It seems that besides the identified phenols, more of this kind of compounds are present in hydrolysates exhibiting inhibitory effects [57,59,63]. To investigate these unknown inhibitors, a non-targeted metabolomics approach needs to be carried out. 

## 4. Non-Targeted Approach. Research Case: Applying Non-Targeted Metabolomics Approach to Study Inhibitors and Their Dynamics in Lignocellulosic Biomass Hydrolysates as Fermentation Media

As discussed in the sections above, in this area of research true non-targeted metabolomics approaches have not yet been completed. Therefore, we here describe an example case of such study based on our own research, illustrating corresponding and differentiating aspects of such as study compared to targeted approaches.

As discussed in the previous section, when used as fermentation media, hydrolysates show toxicity towards fermenting microbes, due to the degradation products of (hemi-) cellulose and lignin. The toxicity varies with different types of hydrolysates, and is mainly determined by the pretreatment-hydrolysis method used, but is also influenced by the biomass type [11,78]. 

Targeted metabolomics has been used to study the toxicity of biomass hydrolysates in fermentation processes by analyzing the composition of (hemi-) cellulose and lignin degradation products. However, it is believed that besides the identified inhibitors, there are still other non-sugar compounds and their derivatives present in biomass hydrolysates that may show toxicity or influence the toxicity of other compounds by synergistic or antagonistic effects. This is because the identified inhibitors alone do not fully explain the toxicity of biomass hydrolysates [57,59,63]. To explore the identity of these unknown compounds, the composition of biomass hydrolysates needs to be studied in a non-targeted manner, alongside the dynamics of these compounds and their effects during fermentation processes. The metabolomics approach introduced in section 2 is adopted to carry out such a study (Figure 2B). In the following sections, the steps of this study are described in more detail. 

### 4.1. Define Research Question

The aim of the study was to identify compounds that (negatively) influence the hydrolysate fermentability through analyzing the composition of different hydrolysates. The corresponding research question was to identify inhibitors in biomass hydrolysates relevant for ethanolic fermentation of *S. cerevisiae*. This question can be differentiated into an experimental and a statistical research question. The experimental question was to determine which non-sugar compounds in hydrolysates are responsible for the hydrolysate toxicity towards microbes in a fermentation process. The statistical question was to determine which of the variables contribute the most to the fermentation performance phenotype(s) (Figure 2B). The variables are the detectable non-sugar compounds in hydrolysates, while the phenotypes were defined to quantitatively describe the fermentation processes. 

### 4.2. Experimental Design

In the next step, experiments were designed to answer the research question. The statistical question was first considered before any wet-lab experiments were designed. The three aspects of the statistical question were (1) selecting statistical model(s), (2) estimating the number of detectable metabolites, and (3) determining how many experiments to be carried out. In our particular case, partial least square (PLS) model was selected, as it provides, as described in section 2, those variables that most closely relate to the phenotypes. To estimate the number of detectable non-sugar compounds present in hydrolysates, the results of previous hydrolysate composition studies were used. As summarized in Table 1, Table 2, there were in total about 10 small carboxylic acids, 5 furans and 60 phenolic compounds identified. We assumed that a similar number of inhibitory compounds in hydrolysates were not yet detected, giving 150 compounds in total. This number was used to determine the number of experiments to be carried out in the experimental design. The non-sugar compounds in biomass hydrolysates are mainly (hemi-) cellulose and lignin degradation products. The formation of these compounds are interrelated, for instance, formic acid is partially formed from HMF, and furfuryl alcohol is the conversion product of furfural [45,79]. As lignocellulosic biomass is consisted of a relatively small number of building-blocks (Figure 1), it was assumed that the above-mentioned 150 detectable compounds present in hydrolysates, represent only 15–20 groups of compounds formed completely independently. For regression models like PLS, the number of experiments is preferred to be larger than the independent variables in the system. Therefore, approximately 20 experiments were to be carried out. 

Knowing that about 20 different experiments were to be conducted, the wet-lab experiments were designed by resolving the following four aspects: (1) generating different experiments, (2) checking the diversity and reproducibility of these experiments, (3) setting up experimental and sampling procedures, and 4) defining phenotypes. 

Different experiments were acquired by conducting batch fermentation with different biomass hydrolysates. These hydrolysates were prepared with various biomass types and different pretreatment-hydrolysis methods [82,83,84]. To obtain about 20 experiments, six biomass types and four pretreatment-hydrolysis methods were selected. The six biomass types were wheat straw, barley straw, corn stover, bagasse, willow wood and oak wood. They represented the most widely used biomass in the category of agriculture residue, sugar industry by-product, and wood [51,62,82,85,86,87]. Straw is the main agriculture residue in Europe, while corn stover is mostly produced in North and South America. Of the four pretreatment-hydrolysis methods, three used enzymatic hydrolysis, and their pretreatment methods included acid, alkali and oxidative treatment. The fourth method used high concentration of sulfuric acid for both pretreatment and hydrolysis [11]. 

**Figure 3:**
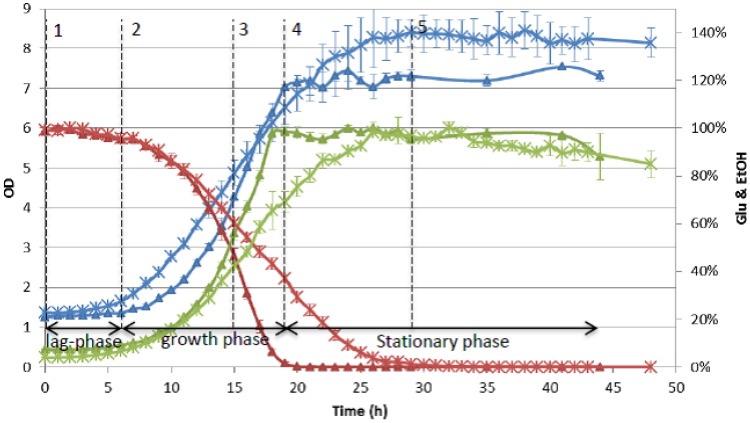
Duplicate fermentation results of the following two hydrolysates: wheat straw-mild alkaline (triangle) and wheat straw-dilute acid (star) (blue: OD, red: glucose percentage, green: ethanol percentage); and illustration of the three fermentation phases and the five selected sample points with wheat straw-mild alkaline fermentation (triangle).

The hydrolysates were first prepared in small volume, *i.e.*, 50 mL, to check their diversity in fermentability by conducting a screen experiment on microtiter plates. This screen experiment confirmed that, as far as growth rate was considered, there was significant diversity among these hydrolysates [11]. Moreover, two hydrolysates were used to examine the reproducibility of batch fermentation. As shown in Figure 3, of both hydrolysates, the fermentation process was clearly presented by both duplicates. Through these pre-experiments, a good basis was formed for the full-scale experiment. 

The full-scale experiment was carried out by fermenting all these hydrolysates individually. These fermentations had a fixed set-up and the same inoculum, so that the difference in fermentation performance was only caused by different hydrolysates [67]. For each fermentation, samples were taken during the whole fermentation process. With these samples biomass formation, glucose and ethanol concentration were measured. These measurements were used to visualize the fermentation process and calculate phenotypes.

Phenotypes are the quantitative description of a fermentation process. In this study, four phenotypes were defined, which were lag-phase, glucose consumption rate, ethanol production rate and ethanol yield (Figure 2B). Lag-phase was a phenotype expressed in hours, which was used to describe the time window before growth starts. Glucose consumption rate and ethanol production rate expressed how quick the microbe grows and how fast the product is produced. Ethanol yield indicated the production efficiency. Each of these phenotypes tackled a different aspect of the fermentation, and together described the whole fermentation process. It should be noted that more phenotypes could be defined, such as growth rate and productivity. However, since the fermentation aspects these phenotypes describe directly relate to one of the four phenotypes defined above, there was little value to include them. 

### 4.3. Sample Selection and Analysis

To analyze the hydrolysate composition during a fermentation process, samples representing the fermentation process were selected. The fermentation process was divided into three different phases based on the phenotypes, namely lag phase, growth phase and stationary phase, see Figure 3. Based on these phases, samples were selected for analysis: three at the beginning of each phase, one at the mid-point of growth phase, and one at the end of stationary phase (Figure 3). In our particular case, these five samples represented the whole fermentation process. 

It was decided to analyze the selected samples with two GC-MS methods for their non-sugar composition, as GC-MS is capable of detecting a broad range of compounds, including several knowns (Table 2). As the compounds of interest in this study are potential inhibitors in biomass hydrolysates, it is important to remove sugars from the fermentation samples. This is mainly because sugars were present in large quantity in those samples, which severely interferes with the detection of non-sugar compounds [49,59]. For this purpose, two sample work-up methods were used, namely, ethyl acetate extraction and ethylchloroformate derivatization. 

Ethyl acetate extraction GC-MS (EA-GC-MS) was adopted from the method described by Heer *et al.* [55]. In this method, the hydrolysate samples were extracted with ethyl acetate (EA), compounds that are apolar, e.g. with aromatic rings, dissolved in EA, while polar compounds, like sugars, remained in the water phase. In this way, sugars were removed from the hydrolysate samples, and the extracted compounds were concentrated. As the solubility of different compounds varies in EA, recovery was a main issue in EA-GC-MS method. Therefore, before analyzing hydrolysates, the recovery of EA method was assessed with several reference compounds. This allowed the estimation of compound recovery in fermentation samples. 

Ethylchloroformate derivatization GC-MS (EC-GC-MS) was adapted to the use for the analysis of biomass hydrolysate samples [67]. The method converts acids to their ethyl ester form through derivatization with ethylchloroformate and extracts the derivatized sample with dichloromethane (DCM). This allowed the detection of carboxylic acids, amino acids, aromatic compounds and furans. The main issue of EC-GC-MS method was the diverse reactivity of different compounds with ethylchloroformate. This may result in detecting compounds present at high concentration with low recovery. The compounds detected by EC-GC-MS partly overlapped but also complemented the ones measured with EA-GC-MS.

From these analyses,“compound lists” will be generated for each method. The initial peak characterization will be done by comparing the mass spectra of these peaks with the mass spectral reference libraries available in our lab. 

### 4.4. Data Analysis and Interpretation

The statistical question of our study is, to determine which variables contribute the most to the selected phenotypes. To answer this question, the two data-sets generated in the previous step will be analyzed by building statistical models. The model to be used is partial least square (PLS), which provides compounds that most closely relate to the four defined phenotypes. To conduct model building, the following aspects need to be carefully studied (1) data preprocessing, (2) model input, and (3) model validation method. 

Based on the property of the acquired data-sets, square-root transformation and autoscaling will be conducted to preprocess the data. These two methods are to reduce the heteroscedasticity and to amplify the variation in the data-sets, respectively [38]. 

To model lag-phase, a data-set containing the first two time-point samples (Figure 3) can be used as model input. This is because lag-phase ends at the second sampling point, and it is assumed that after growth starts, the hydrolysate composition has no influence on lag-phase anymore. To model the other three phenotypes, all five time-point samples are to be used, since the influence of any of the five sampling points on these phenotypes cannot be excluded. 

One way to validate the models is to check their ability of predicting the phenotypes of a new data-set. A so-called double cross validation method is preferred to validate the PLS models in this study, as it evaluates the model quality in a more strict manner [28,88]. 

The modeling results will provide, for each phenotype, a set of compounds that contribute the most to that specific phenotype. The next step will then be to evaluate if these compounds are actually inhibitory to the fermenting microbe. The experimental evaluation of the toxicity of known compounds can be relatively simple. However, to evaluate the toxicity of ‘unknown’ compounds, further compound identification is required. 

## 5. Conclusions

This review illustrated the application of exometabolomics approaches, both targeted and non-targeted, in studying lignocellulosic biomass hydrolysates as fermentation media. Through analyzing the composition of hydrolysates, targeted exometabolomics has been applied to identify inhibitory compounds, improve hydrolysate preparation method, and monitor compound dynamics during detoxification and fermentation process. To further reveal the overall non-sugar composition of various hydrolysates and identify fermentation inhibitors in an unbiased manner, a non-targeted approach was introduced. Its application was demonstrated in our research to identify inhibitors in biomass hydrolysates relevant for ethanolic fermentation of *S. cerevisiae*, emphasizing the essential role of experimental design, phenotype definition, selection of both analytical methods and statistical models in the non-targeted metabolomics approach. 
